# *Alloiococcus otitidis* Forms Multispecies Biofilm with *Haemophilus influenzae*: Effects on Antibiotic Susceptibility and Growth in Adverse Conditions

**DOI:** 10.3389/fcimb.2017.00344

**Published:** 2017-08-02

**Authors:** Chun L. Chan, Katharina Richter, Peter-John Wormald, Alkis J. Psaltis, Sarah Vreugde

**Affiliations:** Department of Surgery, Otolaryngology—Head and Neck Surgery, Adelaide University Adelaide, SA, Australia

**Keywords:** *Alloiococcus otitidis*, *Haemophilus influenzae*, biofilm, polymicrobial biofilm, bacterial interference, antibiotic susceptibility, otitis media, otitis media with effusion

## Abstract

Otitis media with effusion (OME) is a biofilm driven disease and commonly accepted otopathogens, such as *Haemophilus influenzae, Streptococcus pneumonia, and Moraxella catarrhalis*, have been demonstrated to form polymicrobial biofilms within the middle ear cleft. However, *Alloiococcus otitidis* (*A. otitidis*), which is one of the most commonly found bacteria within middle ear aspirates of children with OME, has not been described to form biofilms. The aim of this study was to investigate whether *A. otitidis* can form biofilms and investigate the impact on antibiotic susceptibility and survivability in polymicrobial biofilms with *H. influenzae in vitro*. The ability of *A. otitidis* to form single-species and polymicrobial biofilms with *H. influenzae* was explored. Clinical and commercial strains of *A. otitidis* and *H. influenzae* were incubated in brain heart infusion with and without supplementation. Biofilm was imaged using confocal laser scanning microscopy and scanning electron microscopy. Quantification of biofilm biomass and viable bacterial number was assessed using crystal violet assays and viable cell counting in both optimal growth conditions and in adverse growth conditions (depleted media and sub-optimal growth temperature). Antimicrobial susceptibility and changes in antibiotic resistance of single-species and multi-species co-culture were assessed using a microdilution method to assess minimal bactericidal concentration and E-test for amoxicillin and ciprofloxacin. *A. otitidis* formed single-species and polymicrobial biofilms with *H. influenzae*. Additionally, whilst strain dependent, combinations of polymicrobial biofilms decreased antimicrobial susceptibility, albeit a small magnitude, in both planktonic and polymicrobial biofilms. Moreover, *A. otitidis* promoted *H. influenzae* survival by increasing biofilm production in depleted media and at suboptimal growth temperature. Our findings suggest that *A. otitidis* may play an indirect pathogenic role in otitis media by altering *H. influenzae* antibiotic susceptibility and enhancing growth under adverse conditions.

## Introduction

Since the identification of biofilms within the middle ear of children with chronic otitis media with effusion (OME), OME has been considered a biofilm driven disease (Hall-Stoodley et al., 2006; Thornton et al., 2011). The formation of biofilms is significant, as bacteria within biofilms are conferred protection from environmental, host and chemical stressors, and have an increased antibiotic resistance (Donlan and Costerton, 2002; Fergie et al., 2004). Moreover, bacteria in polymicrobial biofilms have been shown to further reinforce this protection through quorum sensing and the transfer of antibiotic resistance genes between strains (Hackman and Wilkins, 1975; Brook, 1991; Brook and Gilmore, 1993; Armbruster et al., 2010).

Bacteria, classically associated as otopathogens, such as *Haemophilus influenzae, Streptococcus pneumoniae, and Moraxella catarrhalis*, have all been demonstrated to form polymicrobial biofilms within the middle ear cleft (Hall-Stoodley et al., 2006; Thornton et al., 2011). However, there remain biofilm forming bacteria within the middle ear that have yet to be identified (Thornton et al., 2011). One such bacterial species that may potentially be forming biofilms within the middle ear is *A. otitidis. A. otitidis* has frequently been reported as one of the most common bacteria within middle ear aspirates of patients with OME (Chan et al., 2016, 2017). Yet, the potential of *A. otitidis* to form biofilms has yet to be investigated.

In the past, *A. otitidis* had been considered to be commensal of the external auditory canal, yet literature suggests that *A. otitidis* may have pathogenic potential. *In vivo* studies have shown *A. otitidis* to have an immune-stimulatory potential on both myeloid (Himi et al., 2000) and lymphoid (Tarkkanen et al., 2000) cell types and has also been shown to induce OME in a rat model (Tano et al., 2008).

The aim of this study was to investigate whether *A. otitidis* formed biofilms and additionally, whether *A. otitidis* forms polymicrobial biofilms with *H. influenzae*, the most commonly associated bacterial cause of otitis media. Moreover, the effect of polymicrobial biofilms of these two bacteria on antibiotic susceptibility and survival was investigated.

## Materials and methods

### Bacterial strains and handling

*A. otitidis ATCC* 51267 and *H. influenzae* ATCC 33391 type strains were purchased from the American Type Culture Collection (ATCC). Also, two clinical non-typeable *H. influenzae* strains designated NT176 and NT1159, both from middle ear isolates, were obtained from the Department of Molecular and Cellular Biology at the University of Adelaide. *A. otitidis* was subcultured from freezer stocks onto Brain Heart Infusion agar supplemented with 5% defibrinated sheep blood (BHIb) and incubated for 4 days at 37°C at normal atmospheric conditions. *H. influenzae* strains were subcultured in BHI agar supplemented with 10 μg/mL hemin (factor X) and 0.2 μg/mL of β-nicotinamide-adenine-dinucleotide (factor V) (BHIs) and incubated overnight at 37°C in 5% CO_2_. All experiments described below were replicated in biological triplicate.

#### Planktonic growth assays

Bacterial colonies were collected from subcultured bacteria (as described above) with a sterile swab and added to 2 mL of 0.45% sodium chloride. This mixture was adjusted to achieve a bacterial suspension with a McFarland unit of 3.0 for *A. otitidis* and McFarland unit of 1.0 for *H. influenzae*. The bacterial suspension was combined with BHIs or BHIb respectively to a ratio of 1:14 (bacterial solution: media). This was equivalent to a starting suspension of 2.0 × 10^7^ single colony forming units per mL (SCFU/mL) of *A. otitidis*, 1.4 × 10^8^ SCFU/mL of ATCC 33391, 4.2 × 10^7^ SCFU/mL of NT 176, and 4.8 × 10^7^ SCFU/mL of NT 1159. The combination of *A. otitidis* alone, *H. influenzae* alone and in combination were made up 1:1 in BHIs up to 10 mL (5 mL each suspension) and incubated for 24 h. Single colony forming unit (SCFU) assays were used to enumerated viable cell count at 1, 2, 4, 6, 12, and 24 h to determine growth characteristics.

#### Determination of selective plates

The requirement of factor V and X for *H. influenzae* growth is well documented (Musher, 1996); therefore BHI with 5% sheep's blood was used as a selective medium for *A. otitidis* with enumeration at 72 h. *H. influenzae* was enumerated on BHIs agar plates after 24 h after initial testing showed no growth of *A. otitidis* after this time.

### Establishing biofilm

One hundred and eighty microliters of bacterial mixture *(A. otitidis* alone, *H. influenzae* alone and in combination) were made up 1:1 in BHIs (i.e., 90 μL of each bacterial strain suspension), was transferred into each of the wells of a 96 (round) well polystyrene microtitre plate (Grenier Cellstar, Frickenhausen). Control wells contained 90 μL *A. otitidis* with 90 μL of BHIs, 90 μL *H. influenzae* with 90 μL of BHIs or BHIs alone. The 96 well plates were then incubated for 24 h in 5% CO_2_ (3.5 L Oxoid Anaerobic gas jar with Oxoid CO_2_ Gen Sachets, Thermo Scientific, Scoresby, Victoria) at 37°C on a gyro-shaker at 70 rpm.

To assess biofilm formation in depleted media, biofilm assays above were repeated in either BHI only, BHI with factor X only, and BHI with factor V only. Furthermore, culture at 30°C with BHIs was also performed.

### Quantifying biofilm

Biofilm was quantified for each combination of bacteria by two means; crystal violet staining for biofilm volume, and SCFU counting for viable bacterial enumeration.

#### Crystal violet assay

Following biofilm formation in the 96-well plates after 24 h, each well was washed twice in Phosphate buffered saline (PBS) to remove non-adherent cells. Two hundred microliters of 100% methanol was added to each well and incubated at room temperature for 15 min. The methanol was suctioned and wells were allowed to air dry. Two hundred microliters of crystal violet 10%w/w was added to each well and incubated at room temperature for 15 min and allowed to dry overnight. Two hundred and ten microliters of 95% ethanol (w/w) was then added to the wells and incubated at 37°C for 1 h to extract the crystal violet for biofilm quantification. Optical density was read at 595 nm using a plate reader instrument (BMG Labtech, Fluostar Optima, Offenburg, Germany).

#### Colony forming unit assay

After formation of biofilm in the 96-well plates after 24 h, each well was washed twice in PBS. Cells were re-suspended in 100 μL of PBS by sonicating in a sonicator bath (Soniclean 80T, Soniclean Pty Ltd., Australia) for 10 min, followed by pipetting up and down. Bacteria were enumerated on selective plates as described above.

### Biofilm imaging

#### Confocal laser scanning microscopy

Biofilms were formed on eight chamber glass slides by adding 300 μL of bacterial suspension *(A. otitidis* alone, *H. influenzae* alone and in combination 1:1) and incubated for 24 h in 5% CO_2_ at 37°C on a gyro-shaker at 70 rpm. The slides were washed thrice in PBS and were prepared for confocal laser scanning microscopy (CLSM) (Richter et al., 2016). Briefly, biofilms were fixed with 2.5% glutaraldehyde, then stained with SYTO 9 (1 μL/mL) and propidium iodide (1 μL/mL) in PBS (Live/Dead® *Bac*light™ bacterial viability kit, Thermo Fischer Scientific, Inc., Waltham, MA, USA). The slides were examined on a Zeiss LSM700 Confocal Laser Scanning Microscope (Carl Zeiss, Jena, Germany).

#### Scanning electron microscopy

Biofilms in the combinations described above (*A. otitidis* alone, *H. influenzae* alone and in combination) were grown for 168 h on pegs in the Calgary Biofilm Device (Innovotech, Edmonton, Canada) in BHIs (for *H. influenzae* and in combination with *A. otitidis*), and BHIb (*A. otitidis alone*), with media replaced every 24 h. Pegs were washed with PBS, fixed with 2.5% glutaraldehyde and exposed to 1% osmium tetroxide, followed by a dehydration series ranging from 50 to 100% ethanol. The final dehydration step was repeated twice before addition of hexamethyldisilazane to dry and preserve the biofilm structure. Biofilms on pegs were then sputter-coated with 5 nm gold and visualized by scanning electron microscopy (Zeiss Gemini 2, Carl Zeiss, Jena, Germany).

### Antibiotic susceptibility testing

Breakpoints were determined as per EUCAST 2016 guidelines (EUCAST, 2016) and were classified as susceptible (S) or resistant (R) as follows for *H. influenzae:* amoxicillin S ≤ 2 μg/mL, R > 2 μg/mL, ciprofloxacin S ≤ 0.5 μg/mL, R > 0.5 μg/mL. Antibiotic minimum inhibitory concentration (MIC) breakpoints have not been defined for *A. otitidis*. Therefore, guidelines for *S. pneumoniae* were used to interpret the results as previously reported (Ashhurst-Smith et al., 2007): amoxicillin S ≤ 0.06 μg/mL, R ≥ 2 μg/mL, ciprofloxacin S ≤ 0.125 μg/mL, R ≥ 2 μg/mL.

### Minimum bactericidal concentration of biofilm

Modified minimum bactericidal concentration assays were performed in the same combinations as above (Wiegand et al., 2008). Fifty microliters of *H. influenzae* alone, *A. otitidis* alone, or *H. influenzae* and *A. otitidis* together were added to 96-well plates in BHIs and incubated at 37°C in 5% CO_2_ for 24 h. Wells were washed twice in PBS. Serial dilutions of amoxicillin and ciprofloxacin were made up in BHIs, and 100 μL was added to the wells. The plates were incubated for an additional 24 h, and bacteria were enumerated via SCFU counting to establish minimal bactericidal concentrations (MBC).

Further, antibiotic testing was carried out by E-Test® (bioMérieux, SA, Marcy l'Etoile, France) with the above combinations of *H. influenzae* and *A. otitidis*. BHIb agar was utilized for testing of *A. otitidis* alone, and BHIs was used for testing of *H. influenzae* and when *H. influenzae* was in combination with *A. otitidis*. One milliliter of bacterial suspension in 0.45% normal saline (McFarland standard of 1.0 and 3.0 for *H. influenzae* and *A. otitidis* respectively) was added to 30 mL of agar. Plates were incubated in 5%CO_2_ for 24 h when testing *H. influenzae* susceptibility and 96 h for *A. otitidis*. Susceptibility to ciprofloxacin and amoxicillin was tested.

#### Statistical analysis

Pearson's GraphPad v6 (GraphPad Software Inc., California, USA) was used to analyze the data, generate box plots and calculate the statistical tests used. Student's *t*-test (two-tailed, unequal variance) was used to analyze the significance of differences between single colony unit counts and optical densities of biofilm cultures. Data with a *p*-value of 0.05 or less were considered statistically significant.

## Results

### *A. otitidis* planktonic growth and biofilm formation

*A. otitidis* grew both in BHIb and BHIs, with peak growth at 24 h. Single colonies achieved a stable size by day 4 in both media but were larger when grown in BHIb. There were a greater number of viable cells in BHIb than in BHIs at 12 h (BHIb, Mean SCFU/mL [*SD*] = 7.7 × 10^6^, [3.1 × 10^6^] vs. BHIs, 1.0 × 10^6^ [7.5 × 10^5^], *p* = 0.02), 24 h (BHIb, 6.7 × 10^7^ [1.2 × 10^7^] vs. BHIs, 9.0 × 10^6^ [1.0 × 10^6^], *p* = 0.001), and 48 h (BHIb, 3.2 × 10^7^ [1.6 × 10^7^] vs. BHIs, 5.6 × 10^6^ [5.7 × 10^5^], *p* = 0.046) (Supplementary Figure 1). There was no difference in growth when incubated at normal atmosphere or in 5% CO_2_.

**Figure 1:**
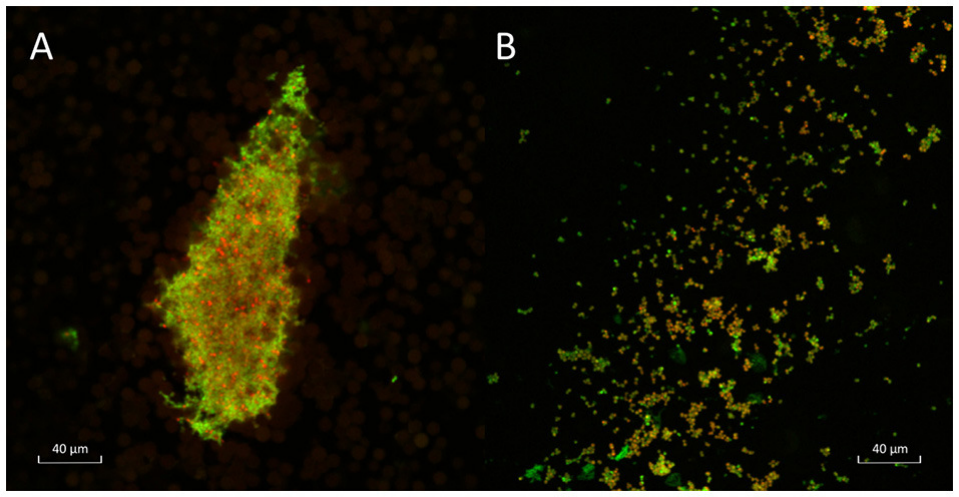
Confocal laser scanning microscopy of *A. otitidis*. Confocal laser scanning microscopy (CLSM) of dense satellite-aggregations of *A. otitidis* in BHIb **(A)** and sparser aggregations in BHIs **(B)** at X63 magnification. Live/Dead Baclight Bacterial Viability staining was used: live cells (with intact cell membranes) stain green and dead or dying cells (with compromised cell membranes) stain red.

CLSM with LIVE/DEAD *Bac*light bacterial viability stains indicated that when grown in BHIb, *A. otitidis* formed dense, satellite-aggregations of cells with extracellular matrix, consistent with biofilm (Figure 1A). When grown in BHIs, cells appeared more dispersed, and aggregations were smaller in size (Figure 1B). Peak viability of cells and biofilm biomass assessed with SCFU and crystal violet assay occurred at 48 h of incubation.

### Co-culture of *H. influenzae* and *A. otitidis* and formation of polymicrobial biofilm

As expected, *H. influenzae* required factors V and X to grow (Evans et al., 1974) and did not grow in BHIb. In co-culture in BHIs, there was no significant change in the number of viable cells or optical density of the broth, of co-cultured *H. influenzae* and *A. otitidis* compared to single-species controls in the first 48 h of growth.

**Figure 2:**
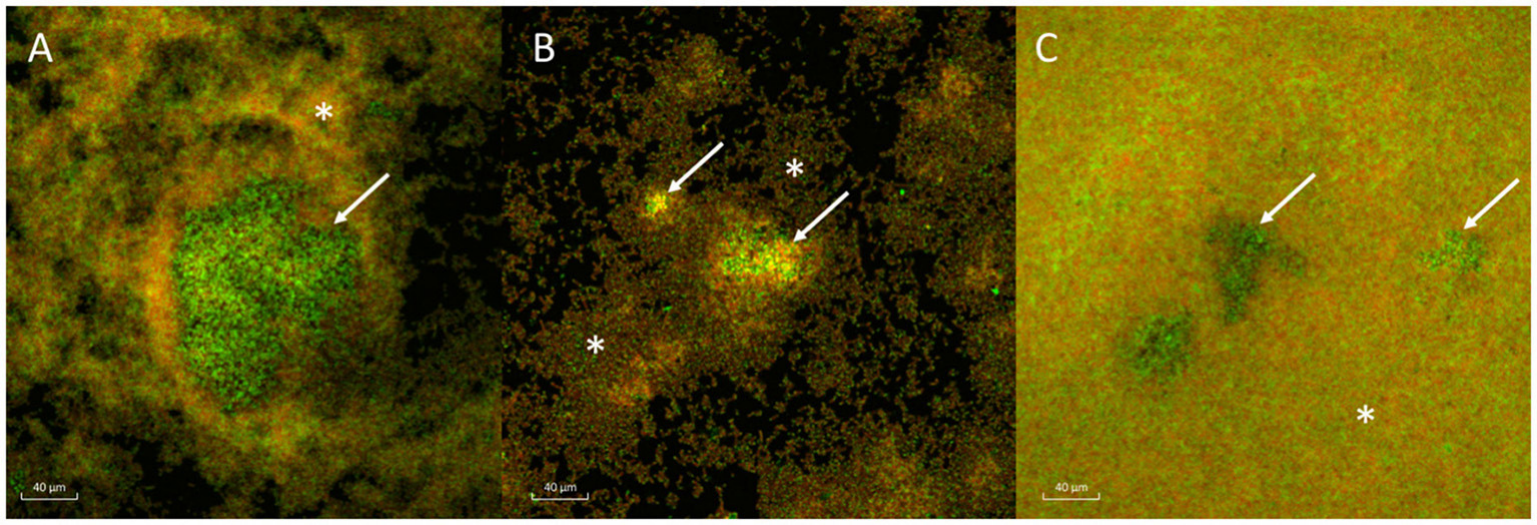
Confocal laser scanning microscopy of *A. otitidis* and *H. influenzae* multispecies biofilm. **(A–C)** CLSM of multispecies biofilm in BHIs at X63 magnification, after LIVE/DEAD *Bac*light Bacterial Viability staining consisting of *A. otitidis* (Green cocci, arrows) and *H. influenzae* (mixed, smaller, orange-green coccobacilli, asterisks). The *H. influenzae* strains shown are NT 1159 **(A)**, NT 176 **(B)**, and ATCC 33391 **(C)**.

**Figure 3:**
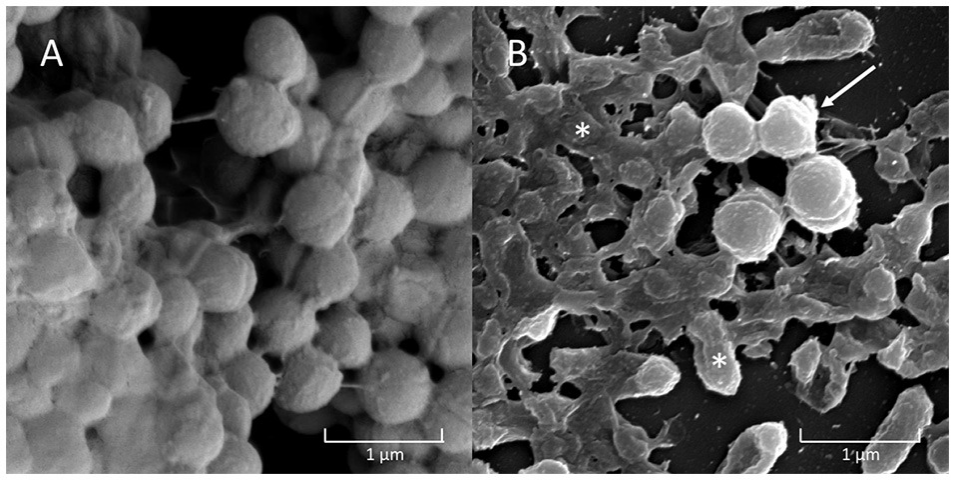
Scanning Electron Microscopy (SEM) of *A. otitidis* and *H. influenzae* multispecies biofilm. *A. otitidis* single species biofilm in BHIb **(A)** and multispecies biofilm in BHIs **(B)** of *A. otitidis* (cocci, arrows) and *H. influenzae* (rods, asterisks).

All three strains of *H. influenzae* formed biofilm in BHIs. When co-cultured with *A. otitidis* in BHIs, polymicrobial biofilm was observed with all three strains (Figure 2). The aggregates of *A. otitidis* biofilm formed in polymicrobial biofilm were reminiscent of those demonstrated when *A. otitidis* was cultured alone in BHIb. *A. otitidis* biofilm (Figure 3A) and polymicrobial biofilms (Figure 3B) were visualized by scanning electron microscopy, where clusters of *A. otitidis* were observed next to *H. influenzae* biofilm.

**Figure 4:**
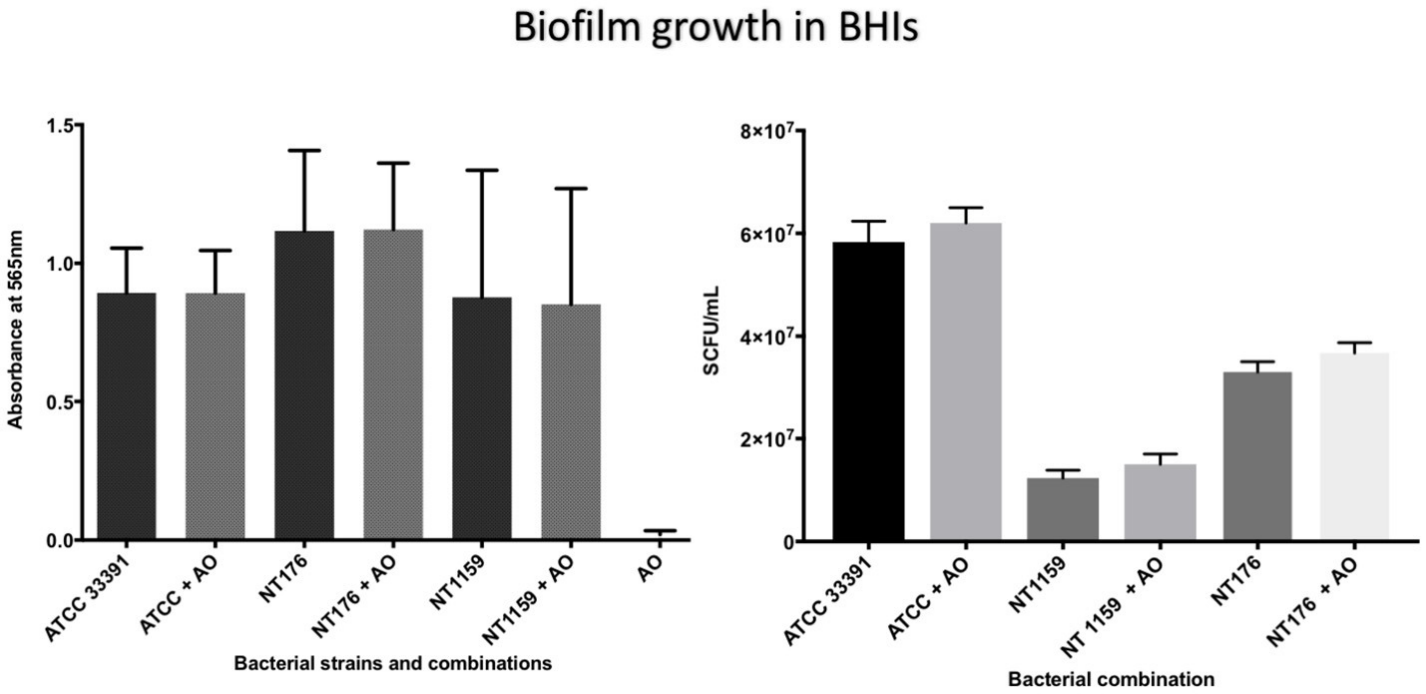
Crystal violet assay and viable cell counts of polymicrobial biofilm in BHIs. Quantification of the three *H. influenzae* (ATCC 33391, NT176, and NT1159) biofilms, alone and in the presence of *A. otitidis* (AO), in reduced brain heart infusion supplemented with Factor V and X. Graphs represent crystal violet staining and absorbance reading at 565 nm **(Left)** with corresponding SCFU counts **(Right)**. Data are presented as means ± *SD* for *n* = 4.

The number of viable *H. influenzae* cells via SCFU counting was similar from mono- or co-cultured biofilm in BHIs. Additionally, total biofilm biomass, as reflected in the results of the crystal violet assays and SCFU counts, was similar for co-culture with all three *H. influenzae* strains (Table 1, Figure 4).

**Table 1:** Viable cell counts of *Haemophilus influenzae* in biofilm when cultured alone or in combination with *Alloiococcus otitidis* and when cultured in different media.

	**ATCC 33391**	**ATCC 33391 + AO**	***P*-value**	**NT 1159**	**NT 1159 + AO**	***P*-value**	**NT 176**	**NT 176 + AO**	***P*-value**
BHI + Factor V and X (BHIs)	5.8 × 10^7^ (2.3 × 10^6^)	6.2 × 10^7^ (1.7 × 10^6^)	0.28	1.2 × 10^7^ (8.8 × 10^5^)	1.5 × 10^7^ (1.2 × 10^6^)	0.14	3.3 × 10^7^ (1.2 × 10^6^)	3.7 × 10^7^ (1.2 × 10^6^)	0.09
BHI + Factor V (Hemin)	2.4 × 10^4^ (8.7 × 10^3^)	1.4 × 10^7^ (2.5 × 10^6^)	0.002^*^	5.2 × 10^3^ (1.5 × 10^2^)	1.7 × 10^6^ (3.2 × 10^5^)	0.006^*^	9.0 × 10^3^ (1.7 × 10^3^)	5.6 × 10^6^ (7.8 × 10^5^)	0.002^*^
BHI + Factor X (NAD)	1.5 × 10^6^ (3.4 × 10^5^)	5.1 × 10^6^ (8.8 × 10^5^)	0.008^*^	1.1 × 10^4^ (2.2 × 10^3^)	3.7 × 10^5^ (2.7 × 10^5^)	0.2529	1.2 × 10^4^ (1.7 × 10^3^)	8.7 × 10^5^ (8.8 × 10^4^)	0.001^*^

*Single colony forming unit enumeration on selective plates (BHIs) of the three strains of H. influenzae (ATCC 33391, NT176, and NT 1159) when cultured alone or co-cultured with A. otitidis (AO). Bacterial combinations were incubated for 24 h in 5% CO_2_ at 37°C on a gyro-shaker. Experiments were conducted in optimal media (BHI + Factor V and X), and reduced media (BHI + Factor V only and BHI + Factor X only). ([Mean SCFU/mL (SD)]*.

**Figure 5:**
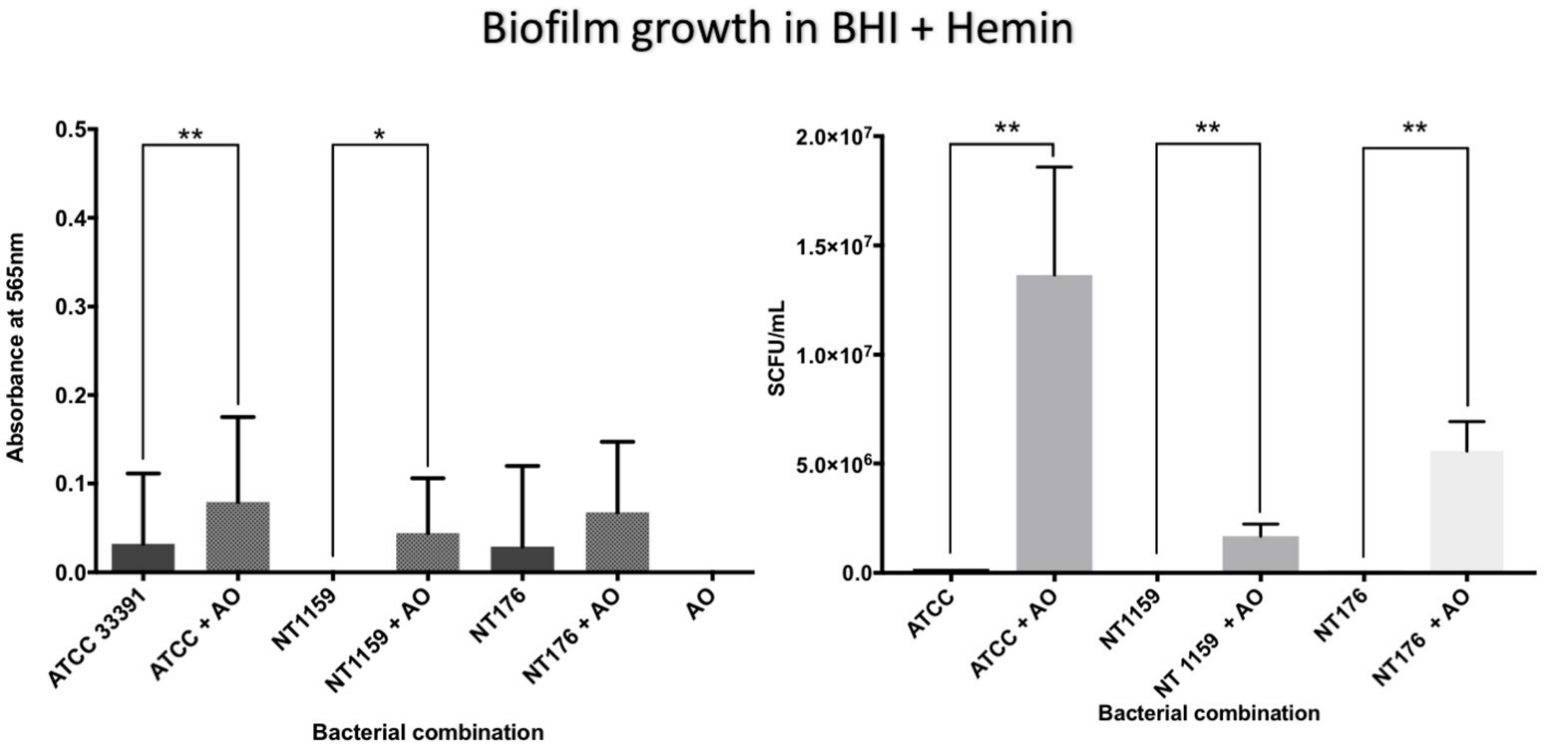
Crystal violet assay and viable cell counts of polymicrobial biofilm in BHI + Hemin. Q Quantification of the three *H. influenzae* (ATCC 33391, NT176, and NT1159) biofilms, alone and in the presence of *A. otitidis* (AO), in reduced brain heart infusion supplemented with Factor X (hemin). Graphs represent crystal violet staining and absorbance reading at 565 nm **(Left)** with corresponding SCFU counts **(Right)**. Data are presented as means ± *SD* for *n* = 3. ^*^p < 0.05, ^**^p < 0.01.

**Figure 6:**
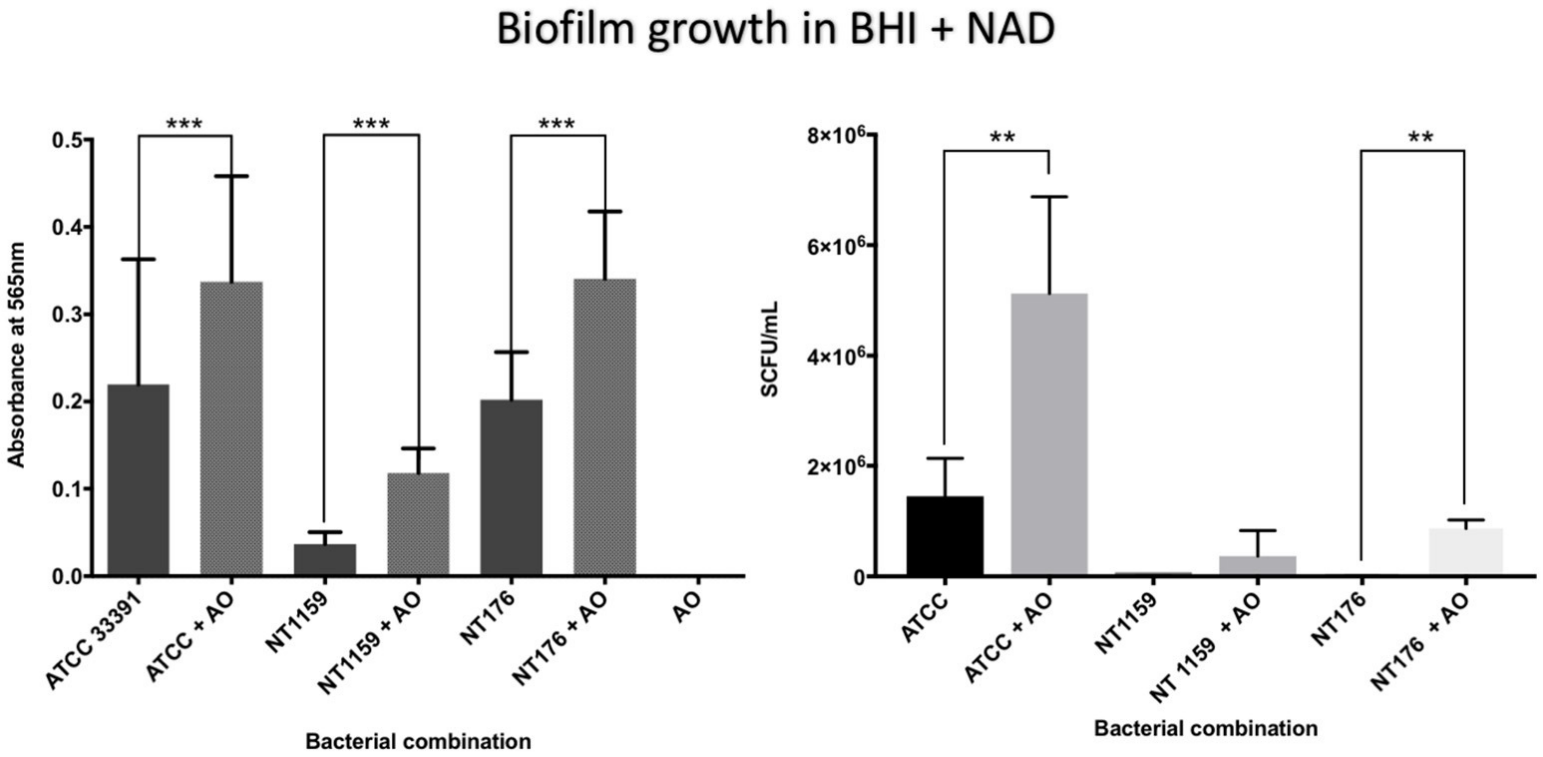
Crystal violet assay and viable cell counts of polymicrobial biofilm in BHIs + NAD. Quantification of the three *H. influenzae* (ATCC 33391, NT176, and NT1159) biofilms, alone and in the presence of *A. otitidis* (AO), in reduced brain heart infusion supplemented with Factor V (nicotinamide adenine dinucleotide - NAD). Graphs represent crystal violet staining and absorbance reading at 565 **(Left)** with corresponding SCFU counts **(Right)**. Data are presented as means ± *SD* for *n* = 3. ^**^p < 0.01, ^***^p < 0.001.

In contrast, when *H. influenzae* was cultured in suboptimal conditions (i.e. BHI in the absence of factor V or X or growth at 30°C), the addition of *A. otitidis* increased the total number of viable *H. influenzae* cells and the total biofilm biomass. No biofilm was demonstrated at 24 h in BHI only, for either *H. influenzae* only, *H. influenzae* with *A. otitidis* or *A. otitidis* only. However, when *H. influenzae* ATCC 33391 was grown in BHI with Factor V (Hemin) and Factor X (NAD), there was a significant increase in optical density for co-cultured biofilm (0.01 vs. 0.08, 95% CI 0.05 to 0.13, *p* < 0.001 and 0.22 vs. 0.34, 95% CI 0.06 to 0.18, *p* < 0.001 respectively) (Figures 5, 6) and viable cell count (Table 1). Similar results were demonstrated for viable cell count (Table 1) and optical density of NT 1159 biofilm (0.001 vs. 0.04, 95% CI 0.01 to 0.08, *p* = 0.007 and 0.2 vs. 0.34, 95% CI 0.09 to 0.18, *p* < 0.001) and likewise for NT 176 biofilm (0.03 vs. 0.07, 95% CI 0.02 to 0.09, *p* = 0.19 and 0.04 vs. 0.12, 95% CI 0.06632 to 0.096, *p* < 0.001) (Figures 5, 6) and viable cell counts (Table 1).

**Figure 7:**
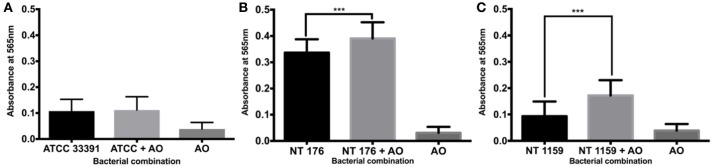
Crystal violet assay of polymicrobial biofilm at 30°C. Quantification of *H. influenzae* ATCC 33391 (ATCC) **(A)**, NT 176 **(B)**, and NT 1159 **(C)** biofilm biomass in the presence or absence of *A. otitidis* biofilm grown for 24 h at 30°C in BHIs. Biofilm biomass was measured using crystal violet staining and absorbance reading at 565 nm. Data are presented as means ± *SD* for *n* = 3. ^***^p < 0.001.

For culture at 30°C, there was a significant increase in biofilm mass demonstrated using the crystal violet assay for the non-typeable *H. influenzae* strains NT 176 and NT 1159 (0.34 SD 0.05 vs. 0.39 SD 0.07, 95% CI 0.03 to 0.08, *p* < 0.001 and 0.09 SD 0.06 vs. 0.17 SD 0.06 CI 0.05 to 0.11, *p* < 0.001) when grown in co-culture with *A. otitidis* (Figure 7).

### Antibiotic susceptibility

#### A. otitidis

*A. otitidis* was found to be susceptible to both amoxicillin and ciprofloxacin by E-test (0.016 and 0.016 μg/mL respectively) (Table 2). In co-culture, there was a change in the MIC of *A. otitidis* in combination with all 3 strains of *H. influenzae*, with a 2.9-fold decrease in susceptibility to amoxicillin (0.016 vs. 0.047 μg/mL).

**Table 2:** Minimum inhibitory concentrations (MIC) and Minimum Bactericidal Concentration (MBC) for *A. otitidis* (AO) planktonic cells and biofilm alone and in combination with *H. influenzae* (ATCC 33391, NT176, and NT 1159).

**Bacterial combinations**	**MIC (μg/mL) (Planktonic)**	**MBC (μg/mL) (Biofilm)**
**AMOXICILLIN**		
*A. otitidis* (AO)	0.016 (S)	0.125
AO + ATCC 33391	0.047 (S)	0.125
AO + NT 176	0.047 (S)	0.06
AO + NT 1159	0.047 (S)	0.125
**CIPROFLOXACIN**		
AO	0.016 (S)	0.25
AO + ATCC 33391	0.016 (S)	0.25
AO + NT 176	0.012 (S)	0.125
AO + NT 1159	0.016 (S)	0.25

*MIC was assessed by E-test with BHIb agar, of A. otitidis monospecies or in co-culture with H. influenzae (ATCC 33391, NT176, and NT1159 strains). All strains were susceptible (S) to amoxicillin and ciprofloxacin in planktonic form (MIC ≤ 0.06 μg/mL and S ≤ 0.125 μg/mL respectively). MBC of A. otitidis monospecies or in co-culture with H. influenzae was determined by SCFU counting*.

For ciprofloxacin, when *A. otitidis* was co-cultured in combination with *H. influenzae* strain NT176, the MIC fell from 0.016 to 0.012 μg/mL. *H. influenzae* strains NT 1159 and ATCC 33391 did not alter the MIC of *A. otitidis*.

In the microdilution assays, the MBC of *A. otitidis* for amoxicillin and ciprofloxacin was 0.125 and 0.25 μg/mL respectively. With the addition of *H. influenzae* strain NT 176, the MBC decreased for both amoxicillin and ciprofloxacin (0.06 and 0.125 μg/mL respectively). *H. influenzae* strains NT 1159 and ATCC did not influence MBC.

#### H. influenzae

*All three H. influenzae* were susceptible to both amoxicillin and ciprofloxacin (Table 3). Again, co-culture affected the susceptibility in some combinations. In co-culture with *A. otitidis*, there was a relative decrease in the susceptibility of NT 176 to amoxicillin (MIC = 1.5 vs. 2.0 μg/mL). There was no change to the MICs of strains ATCC 33391 or NT 1159.

**Table 3:** Minimum inhibitory concentrations (MIC) and Minimum Bactericidal Concentration (MBC) for *H. influenzae* (HI) (ATCC 33391, NT176, and NT1159 strains) planktonic cells and biofilm.

***H. influenzae* strain**	**MIC HI alone**	**MIC with *A. otitidis* in co-culture (μg/mL)**	**MBC HI alone**	**MBC with *A. otitidis* in co-culture (μg/mL)**
**AMOXICILLIN**				
ATCC *33391*	1.0 (S)	1.0 (S)	32	32
NT 176	1.5 (S)	2.0 (S)	32	64
NT 1159	1.0 (S)	1.0 (S)	32	32
**CIPROFLOXACIN**				
ATCC *33391*	0.008 (S)	0.012 (S)	0.1	0.1
NT 176	0.012 (S)	0.012 (S)	0.05	0.1
NT 1159	0.008 (S)	0.012 (S)	0.023	0.023

*Minimum inhibitory concentrations (MIC) and Minimum bactericidal concentration (MBC) for H. influenzae (HI) (strains ATCC 33391, NT 176, and NT 1159) in planktonic and biofilm antibiotic susceptibility respectively. MIC was assessed by E-test method with BHIs agar of H. influenzae monospecies or in co-culture with A. otitidis. All strains were susceptible (S) to amoxicillin and ciprofloxacin in planktonic form (MIC ≤ 2 μg/mL and ≤ 0.5 μg/mL respectively). MBC of H. influenzae monospecies or in co-culture with A. otitidis was determined by SCFU counting*.

For ciprofloxacin, when in co-culture with *A. otitidis*, there was a relative decrease in susceptibility for strains ATCC 33391 (MIC = 0.008 vs. 0.012 μg/mL) and NT 1159 (MIC = 0.008 vs. 0.012 μg/mL). There was no change in MIC for strain NT 176.

## Discussion

This is the first study to our knowledge to demonstrate that *A. otitidis* forms biofilm *in vitro*. Additionally, we demonstrated that *A. otitidis* and *H. influenzae* form polymicrobial biofilms and that once in this form, these bacteria develop traits that promote bacterial survival and resistance to treatment.

*A. otitidis* is the most prevalent bacterial species found in patients with non-purulent OME (20–40%) (Leskinen et al., 2004; Guvenc et al., 2010; Marsh et al., 2012; Chan et al., 2016, 2017). In the past, *A. otitidis* has been considered a commensal of the external ear canal (Frank et al., 2003; Tano et al., 2008). However, consensus on the role of *A. otitidis* remains divided, as *A. otitidis* has been shown to have pathogenic traits including eliciting immune responses *in vitro* (Himi et al., 2000; Tarkkanen et al., 2000; Harimaya et al., 2005, 2007a,b, 2009), invading intracellularly (Faden and Dryja, 1989) and having been identified as a pathogen in device related (Marchino et al., 2013) and chronic infection (Cakar et al., 2013). Utilizing culture-independent 16S rRNA sequencing, our department confirmed that *A. otitidis* and *H. influenzae* are the two most common bacteria demonstrated in children with OME and that the external ear canal may act as a reservoir for the middle ear (Chan et al., 2017, 2016). Furthermore, phylogenetic analysis from these studies demonstrated an inverse relationship in relative abundance between *A. otitidis* and *H. influenzae*, suggesting the potential for bacterial interaction in these patients.

In showing that *A. otitidis* forms biofilm, we have added to the evidence that this species of bacteria may play a role in the pathogenesis of OME. OME appears to be a biofilm driven disease and the ability of *A. otitidis* to form biofilm is consistent with this perception (Hall-Stoodley et al., 2006). The life cycle of biofilms closely resembles the natural history of OME, which is characterized by frequent spontaneous resolution, but also by high recurrence rates and persistence of bacteria despite antibiotic treatment. While in biofilm form, the bacteria may cause low grade subacute inflammation and thus perpetuate middle ear effusion (Stewart and Costerton, 2001). However, through planktonic shedding of the biofilm an acute infection with an associated intense inflammatory response can result. Additionally, the formation of biofilm has been shown to provide bacteria a survival and persistence advantage against not only environmental (e.g., fluctuations in temperature, oxygen tension, pH) and chemical (e.g., antibiotics) stressors but also against phagocytosis and humoral immunity (Donlan and Costerton, 2002; Fergie et al., 2004).

In the setting of OM, biofilms were first demonstrated in the middle ear in an animal model of OM (Post, 2001; Ehrlich et al., 2002). Since that time, middle ear biofilm has been shown in patients with chronic suppurative OM, cholesteatoma (Park et al., 2009), tympanostomy tubes (Wang et al., 2014), and also free floating within the middle ear aspirates of children with OME (Van Hoecke et al., 2016). In addition, biofilm has been demonstrated covering the middle ear mucosa (MEM) in the majority of children with OME in two separate studies (Hall-Stoodley et al., 2006; Thornton et al., 2011). Interestingly, one of these studies (Thornton et al., 2011) noted that “a significant proportion” of MEM biofilm samples had bacteria not identifiable by the Fluorescent *In Situ* Hybridization (FISH) probes they used. Given that *A. otitidis* forms biofilm and its high relative abundance within the middle ear cleft of OME, *A. otitidis* may represent a portion of these unidentifiable bacteria. Further experiments using *A. otitidis* specific probes will be needed to confirm this hypothesis.

Another significant finding of this study was that *A. otitidis* and *H. influenzae* co-existed within polymicrobial biofilms. As discussed above, multispecies biofilm is prevalent within the middle ear cavities of children with OME and has been associated with bacterial growth advantage and greater resistance than in single species biofilms (Armbruster et al., 2010; Weimer et al., 2010). Consistent with this we found that the presence of *A. otitidis* promoted *H. influenzae* biofilm growth at suboptimal growth temperatures and in the absence of critical nutrients (factors X and V). In the setting of OME, polymicrobial biofilm formation with *A. otitidis* may thus result in the persistence of *H. influenzae* in biofilm during times of adverse growth conditions. Yet, when conditions are more conducive to growth, *H. influenzae* may then re-emerge in planktonic form and potentially resulting acute infection.

In addition, whilst single species biofilm formation is known to provide increased antibiotic resistance (Stewart and Costerton, 2001; Donlan and Costerton, 2002), polymicrobial biofilm is thought to confer even greater protection from host defenses and antimicrobials(Hackman and Wilkins, 1975; Brook, 1991; Brook and Gilmore, 1993; Armbruster et al., 2010). Some of the recognized mechanisms behind increased resistance in polymicrobial biofilm includes: increased adaptation via quorum sensing, upregulation and transfer of antibiotic resistance genes and increased levels of mutations in antibiotic target molecules (Høiby et al., 2010). In our study, we found that antibiotic susceptibility effects were strain specific, and whilst of small magnitude, resulted in increased antibiotic resistance of investigated *H. influenzae* strains in polymicrobial co-culture. In addition, we propose that the antibiotic effects demonstrated are β-lactamase independent, given that *A. otitidis* has not proven to be a β-lactamase producer (Bosley et al., 1995).

A limitation of this study was the small number of bacterial strains investigated, but we believe that these results highlight an area for potential further research. Whilst only one strain of *A. otitidis* was utilized, due to limitations in obtaining clinical isolates, both commercially available type strains and clinical strains of *H. influenzae* were investigated. Further studies, both *in vitro* and *in vivo*, exploring the ability for the formation of polymicrobial biofilms with clinical isolates of *A. otitidis* and their interactions with *H. influenzae* and other classically accepted otopathogens, such as *M. catarrhalis* and *S. pneumoniae*, will likely provide further insight into the role of *A. otitidis* in the pathogenesis of OME.

## Conclusion

In this study, we have described another potential role that *A. otitidis* plays in the pathogenesis of OME. We demonstrated that *A. otitidis* forms both single- and multi- species biofilm with *H. influenzae*. In addition, when in polymicrobial biofilm, *A. otitidis* can promote *H. influenzae* growth and survival by increasing biofilm production in adverse growth conditions and by altering antimicrobial resistance.

## Author contributions

CC: study design, experimentation, data collection, confocal imaging, data analysis, manuscript write up, manuscript review. KR: study design, SEM imaging, manuscript write up, manuscript review. PW, AP, and SV: study design, data analysis, manuscript review.

### Conflict of interest statement

PW receives royalties from Medtronic, Integra, and Scopis and is a consultant for Neilmed. AP is a consultant for ENT technologies, a consultant for Aerin Medical devices and is on the speakers bureau for Smith & Nephew. The other authors declare that the research was conducted in the absence of any commercial or financial relationships that could be construed as a potential conflict of interest.
