# Supplementary material for: Alloiococcus otitidis Forms Multispecies Biofilm with Haemophilus influenzae: Effects on Antibiotic Susceptibility and Growth in Adverse Conditions
Source: Front Cell Infect Microbiol. 2017 Aug 2;7:344. doi: 10.3389/fcimb.2017.00344 (PMC5539592; doi:10.3389/fcimb.2017.00344)
Supplement: Supplementary file 1 [file DataSheet1.docx]

**Supplement 1 *A. otitidis* growth curve:** Single colony forming units per mL (SCFU/mL) of *A. otitidis* when incubated in Brain Heart Infusion with 5% sheep blood (BHIb) (Solid, dark grey columns) and BHI agar supplemented with 10μg/mL hemin (factor X) and 0.2μg/mL of β-nicotinamide-adenine-dinucleotide (factor V) (BHIs) (Checked column) when incubated over a 48 hour period. *, p < 0.05, **, p < 0.01.
