# Supplementary figures and images for: Alloiococcus otitidis Forms Multispecies Biofilm with Haemophilus influenzae: Effects on Antibiotic Susceptibility and Growth in Adverse Conditions
Source: Front Cell Infect Microbiol. 2017 Aug 2;7:344. doi: 10.3389/fcimb.2017.00344 (PMC5539592; doi:10.3389/fcimb.2017.00344)

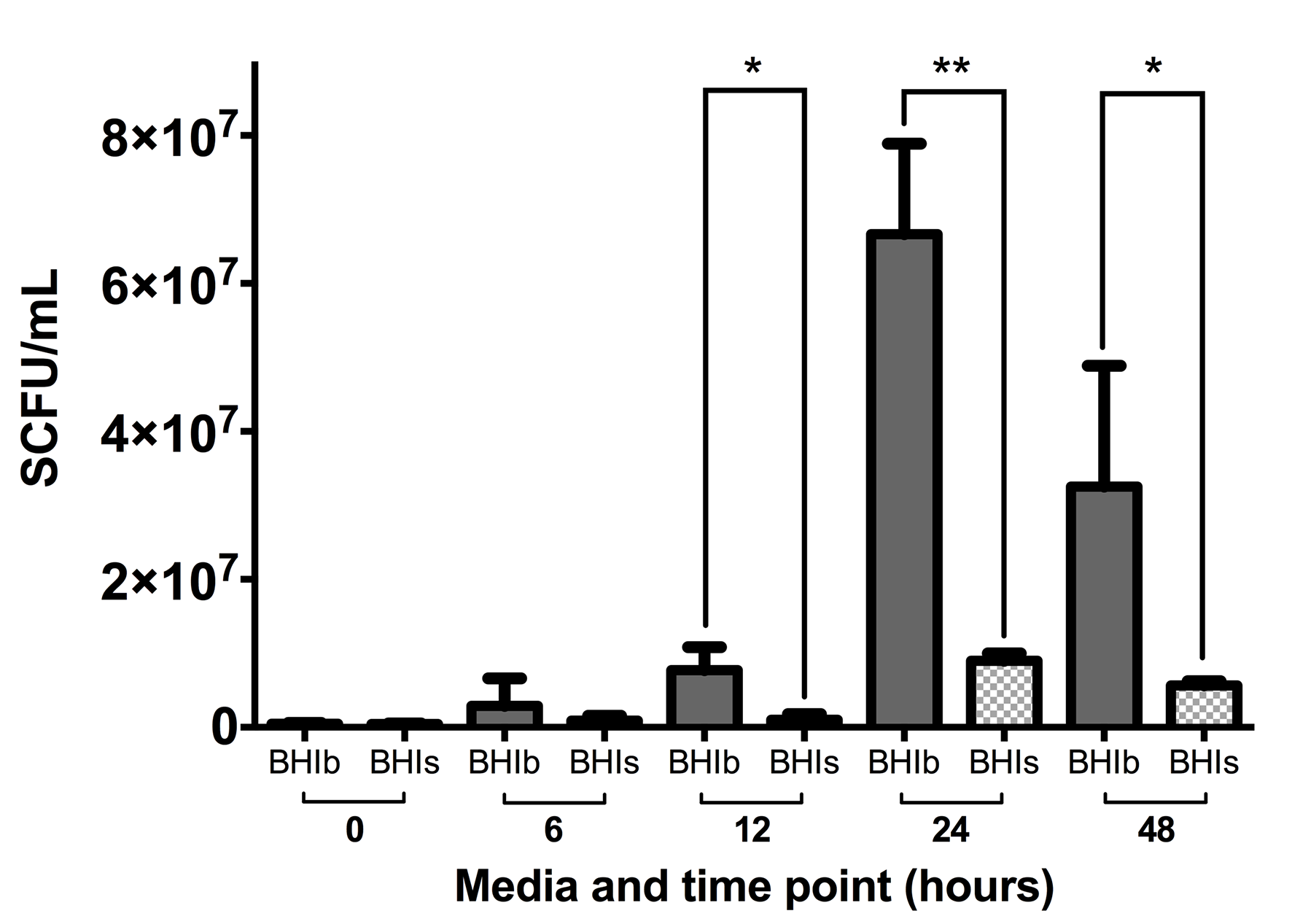

Supplement: Supplementary file 2 [file Image1.TIFF]
